# Does Invasive Mammal Exclusion Restore an Ecosystem Function Mediated by Invertebrates?

**DOI:** 10.1002/ece3.73053

**Published:** 2026-02-11

**Authors:** Corinne Watts, Christopher K. Woolley, James Arbuckle, Scott Bartlam, Joanna K. Carpenter

**Affiliations:** ^1^ Bioeconomy Science Institute Hamilton New Zealand; ^2^ Zealandia Te Māra a Tāne, Karori Wellington New Zealand; ^3^ Te Herenga Waka Victoria University of Wellington Kelburn Parade Wellington New Zealand; ^4^ Bioeconomy Science Institute Dunedin New Zealand

**Keywords:** ecosanctuary, litter decomposition, māhoe, mammal eradication, New Zealand

## Abstract

The removal or exclusion of invasive mammals is a crucial strategy for protecting vulnerable biodiversity, particularly on islands. While eliminating invasive mammals has been shown to increase native species abundance and diversity, it remains unclear whether the ecosystem functions those species provide are also being restored. In particular, the restoration of ecosystem functions performed by invertebrates, such as litter decomposition, pollination and herbivory, is poorly understood. We test whether the removal of invasive mammals restores invertebrate‐mediated decomposition by measuring decomposition of a common native tree (*Melicytus ramiflorus*) within three paired ecosanctuary (all invasive mammals eradicated except mice) and non‐ecosanctuary sites across Aotearoa/New Zealand. Results demonstrated that ecosanctuaries had increased decomposition compared to non‐ecosanctuaries, but this was dependent on litterbag mesh type. Coarse mesh (which allowed access by some larger meso‐ and macrofauna) had greater decomposition in ecosanctuaries compared to non‐ecosanctuaries, but fine mesh had similar decomposition across both site types. Based on other studies of invertebrate diversity inside and outside ecosanctuaries, we suggest this result is largely due to more diverse and abundant detritivore communities within ecosanctuaries. We also detected fine‐scale differences in vegetation composition between ecosanctuaries and non‐ecosanctuaries, probably due to the exclusion of invasive browsing mammals from ecosanctuaries, which also may have influenced decomposition. Our findings suggest that the eradication of invasive mammals can restore an important ecosystem function driven by invertebrates, and we recommend that future studies examine whether other critical invertebrate‐mediated functions (e.g., predation and pest control, pollination) are also being restored.

## Graphical abstract

Excluding invasive mammals is key to protecting native biodiversity, but it is unclear if their removal also restores ecosystem functions, especially those provided by invertebrates, such as decomposition, pollination and herbivory. Fenced ecosanctuaries on mainland New Zealand, which exclude most introduced mammals except mice, offer a unique opportunity to test this. Fenced ecosanctuaries generally have faster invertebrate‐driven litter decomposition compared with non‐ecosanctuary sites, suggesting restoration of this fundamental ecosystem function.

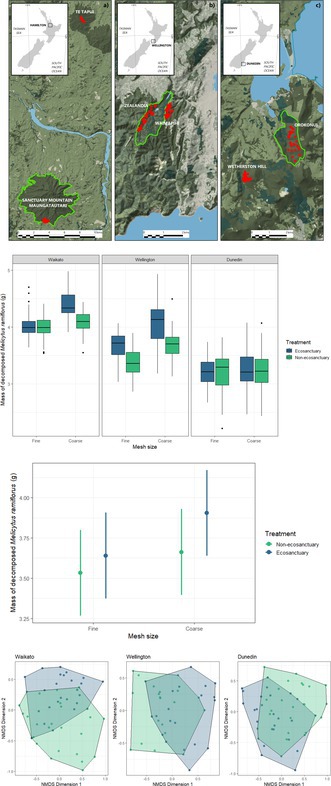

## 1 Introduction

Globally, invasive mammals are one of the leading threats to biodiversity (Doherty et al. 2016; McCreless et al. 2016). Eradication of these mammals (most frequently *Rattus* spp., ungulates and *Felis catus* ) has therefore been an increasingly common strategy used to protect vulnerable threatened species and restore ecosystems (Spatz et al. 2022). Outcome monitoring has generally confirmed that invasive mammal eradications lead to increases in native species abundance and diversity (Binny et al. 2021; Jones et al. 2016). However, the goals of restoration projects are increasingly being expanded beyond the conservation of species themselves to include the interactions and processes carried out by those species, and it is less clear whether these are also reinstated by the removal of invasive mammals (Kaiser‐Bunbury et al. 2010). Several studies have demonstrated that mutualistic ecosystem functions carried out by birds (e.g., pollination and seed dispersal) have been restored or enhanced by the eradication of invasive mammals (Kaiser‐Bunbury et al. 2010), but ecosystem processes mediated by other taxa are still under‐studied.

Invertebrates, particularly large‐bodied terrestrial species, are often affected by predation and habitat modification by invasive mammals (St Clair 2011). Certain groups of terrestrial invertebrates have been shown to increase following the removal of mammalian predators (Chen et al. 2022; Vergara et al. 2021; Watts et al. 2020). However, it is less clear how these changes flow into the many pivotal ecosystem processes carried out by invertebrates, such as litter decomposition and nutrient cycling, pollination, herbivory, seed dispersal and predation (Brockie 1992). Invertebrates play a particularly crucial role in litter decomposition, where plant biomass that enters the dead organic pool as leaf litter must be decomposed before the nutrients are cycled back into the soil and reabsorbed by plants (Swift et al. 1979; Hättenschwiler et al. 2005). This involves a complex process of chemical and physical breakdown mediated by decomposers (bacteria and fungi) and detritivores (animal consumers of dead matter; Bardgett 2005; Gessner et al. 2010). The latter group encompasses invertebrates, ranging in size from microscopic litter mites to large earthworms and snails, which accelerate decomposition by fragmenting plant material, increasing surface area for microbial colonisation and modifying litter chemistry (Lavelle et al. 1997; Coleman et al. 2004).

Invasive mammals can alter decomposition processes through multiple complex pathways (Wardle, Barker, Bonner, and Nicholson 2001; Fukami et al. 2006). Both invasive mammalian herbivores, such as deer and feral goats ( *Capra hircus* ), and invasive predators, such as rats, can directly reduce populations of macrofaunal detritivores, through trampling and physical disturbance by ungulates or through direct predation by mammalian predators (Wardle et al. 2004; Fukami et al. 2006; Mulder et al. 2011). These reductions in detritivore abundance can subsequently slow litter breakdown and alter nutrient cycling processes within invaded ecosystems (Allison and Vitousek 2004; Wardle et al. 2012). For example, predation by invasive rodents has been shown to greatly reduce the densities of detritivorous invertebrates such as Lepidoptera and Gastropoda, which could lead to slower rates of litter decomposition (Crafford 1990; Towns et al. 2009; Angel et al. 2009). Studies on northern Aotearoa/New Zealand (hereafter New Zealand) islands have also shown that predation by invasive rats on indigenous seabirds, which act as critical ecosystem engineers, can have cascading effects on below‐ground processes related to decomposition (Fukami et al. 2006; Towns et al. 2009; Wardle et al. 2009). Invasive mammalian herbivores can also affect litter decomposition processes by altering leaf litter quality, composition and depth. However, these processes are complex and often difficult to study (Bardgett and Wardle 2010).

New Zealand's native fauna and flora largely evolved in the absence of mammalian predators and competitors, producing native ecosystems sensitive to the mammal invasion that accompanied human arrival in New Zealand (Daugherty et al. 1993). To minimise these impacts and restore native ecosystems, New Zealand has become a world leader in invasive mammal eradication (Russell and Broome 2016) and is therefore an ideal context for testing whether the removal and exclusion of invasive mammals restores ecosystem processes such as litter decomposition. After successfully removing invasive mammals from many islands surrounding New Zealand, conservationists also began to eradicate mammals from fenced ecosanctuaries on the mainland (North and South Islands, Stewart Island/Rakiura) of New Zealand (Burns et al. 2012; Bellingham et al. 2010). In New Zealand, ecosanctuaries are defined as ‘a project larger than 25 ha implementing multi‐species, pest mammal control for ecosystem recovery objectives, and with substantial community involvement’ (Innes et al. 2019). Comparable conservation strategies occur internationally, although different terminology is used. For example, in Australia, such sites are often referred to as *mainland islands*, *fenced reserves* or *predator‐free enclosures* (Hayward et al. 2014; Legge et al. 2018), while in Hawaiʻi, similar projects are commonly described as *predator‐proof fenced refuges* or *conservation fences* (Young et al. 2013). There are now 14 of these fenced ecosanctuaries within New Zealand, which exclude both invasive ungulates (e.g., red deer [ *Cervus elaphus* ], feral goats) and predators (e.g., mustelids [*Mustela* spp.], rats and brushtail possums [ *Trichosurus vulpecula* ]) to conserve indigenous biodiversity (Innes et al. 2019). These ecosanctuaries provide an ideal natural experiment (defined as ‘observing conditions that resemble experimental comparisons even though the intervention is not controlled’; Diamond 1983; Paine 2010) to test whether the removal of all invasive mammals (with the exception of the house mouse [ *Mus musculus* ]) restores a key ecosystem process mediated by invertebrates. While replication and treatment assignment cannot be fully controlled in natural experiments, the approach enables inference at spatial and ecological scales that are not achievable in manipulative experiments. In addition, although decomposition is fundamental to ecosystem functioning, it has not previously been assessed in New Zealand ecosanctuaries.

We expected that the invertebrate‐mediated decomposition of a common native tree (māhoe [*Melicytus ramiflorus*]) would be more rapid within three ecosanctuaries compared to paired non‐ecosanctuary sites with comparatively minimal mammal control. Specifically, we hypothesised that decomposition within fine mesh bags (which allow access by bacteria and fungi, but not meso‐ or macro‐invertebrates that are > 1 mm in diameter) should be similar between ecosanctuaries and non‐ecosanctuaries, but that decomposition in the coarse mesh bags would be higher in ecosanctuaries compared to non‐ecosanctuaries. We assumed this would be due to the coarse mesh allowing access to some larger meso‐ and macro‐invertebrates, which are expected to be more abundant and diverse at ecosanctuaries (as found by Chen et al. 2022; Vergara et al. 2021; Watts et al. 2014, 2020).

## 2 Methods

### 2.1 Study Sites

**FIGURE 1:**
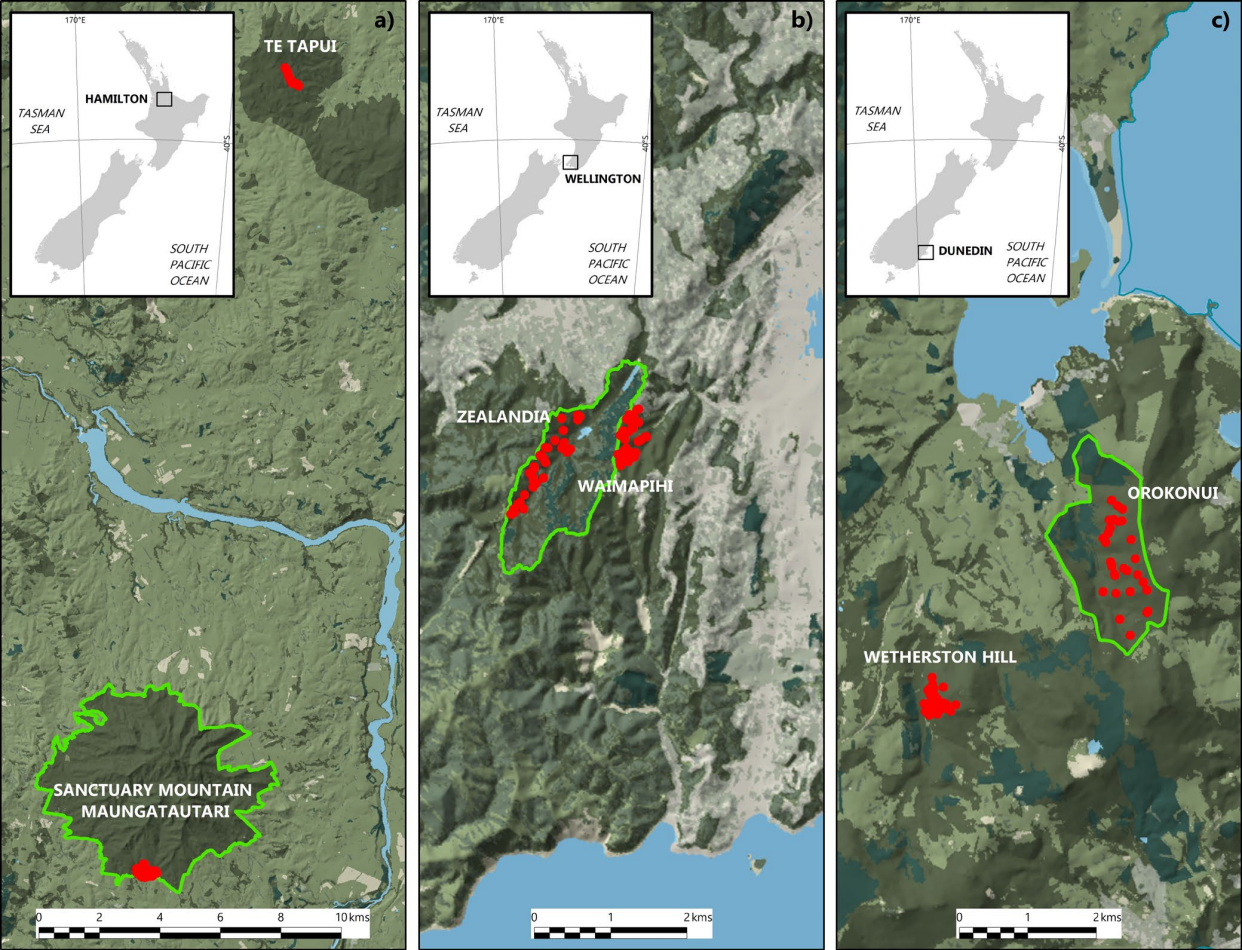
Locations of the three ecosanctuary and non‐ecosanctuary paired sites across New Zealand: (a) Sanctuary Mountain Maungatautari and Te Tapui, Waikato; (b) Zealandia and Waimapihi Reserve, Wellington; and (c) Orokonui and Wetherston Hill, Dunedin. Red circles indicate plots where litter bag plots were placed. Green lines around each ecosanctuary denote the fenced boundary.

We measured litter decomposition at six forested sites across the North and South Islands of New Zealand. We selected three fenced ecosanctuary sites: Te Tūī a Tāne (the southern exclosure) at Sanctuary Mountain Maungatautari (hereafter SMM) near Hamilton in the Waikato, Zealandia Te Māra a Tāne (hereafter Zealandia) in Wellington and Orokonui Ecosanctuary—Te Korowai o Mihiwaka (hereafter Orokonui) in Dunedin, and paired them with nearby non‐ecosanctuary sites (Te Tapui Scenic Reserve in the Waikato (hereafter Te Tapui), Waimapihi Reserve in Wellington, and Wetherston Hill in Dunedin) with comparatively limited mammal control (Figure 1; Table 1). Each of the three ecosanctuaries have eradicated all introduced mammal species, with the exception of mice at Zealandia and Orokonui Ecosanctuary. At the time of the study the unmanaged forests had varying levels of invasive mammal control, but mammals were still present in all of them, particularly possums, rats, mustelids, rabbits ( *Oryctolagus cuniculus* ) and cats (Table 1). Non‐ecosanctuary sites were matched as closely as possible to the ecosanctuary sites by vegetation type, elevation, landform and aspect (Table 1). We also undertook additional temperature measurements across the sites to ensure temperature was well‐matched between the paired sites (see below). Although ideally we would have used a statistical method like propensity score matching (Ramsey et al. 2019) to select non‐treatment sites, the paucity of similar sites (i.e., with the same vegetation type, elevation, landform and aspect) in the vicinity of the ecosanctuaries meant that there were not enough options to require a more quantitative approach.

**TABLE 1:** Attributes of the three fenced ecosanctuary sites and paired nearby non‐ecosanctuary sites used in this study.

Characteristic	Sanctuary Mountain Maungatautari	Te Tapui Scenic Reserve	Zealandia Te Māra a Tāne	Waimapihi Reserve	Orokonui Ecosanctuary	Wetherston Hill
Treatment type	Ecosanctuary	Non‐ecosanctuary	Ecosanctuary	Non‐ecosanctuary	Ecosanctuary	Non‐ecosanctuary
Region	Waikato	Waikato	Wellington	Wellington	Dunedin	Dunedin
Number of days litter bags deployed	230	225	189	190	192	186
Elevation range of sampled points (m asl)	300–360	300–360	180–300	120–280	160–340	100–320
Mean temp (°C)	15.4 (3.2)	15.4 (3.1)	14.4 (2.6)	15.2 (2.7)	12.1 (3.5)	12.3 (3.5)
Mean relative humidity (%)	92.6 (6.8)	91.8 (7.5)	93.2 (5.3)	90.8 (7.3)	85.6 (16.2)	86.6 (15.5)
Dominant forest cover	Tawa, māhoe and silver fern forest	Tawa, māhoe and silver fern forest	Māhoe, *Coprosma* and broadleaf forest	Māhoe, *Coprosma* and broadleaf forest	Kānuka, *Fuchsia*, kāpuka and māhoe forest	Kānuka, *Fuchsia*, kāpuka and māhoe forest
Fence completion year	2006	NA	1999	NA	2007	NA
Mammal control	Eradication of all mammals	Possum control every 2–4 years	Eradication of all mammals except mice	Moderate possum and rat control	Eradication of all mammals except mice	None
Latitude/longitude	−38.029, 175.570	−37.814, 175.611	−41.292, 174.750	−41.300, 174.752	−45.775, 170.603	−45.787, 170.564

*Note:* Mean temperature (temp) and relative humidity were recorded from our dataloggers from November 2023 to March 2024. Standard deviations are given in parentheses.

### 2.2 Litter Decomposition and Site Characteristics

We determined rates of litter decomposition by measuring mass loss of māhoe leaves in litter bags. Māhoe is a small, widespread, abundant tree from the Violaceae family, present at all six sites. Live, ‘healthy‐looking’ leaves were harvested (i.e., hand picked) from up to 10 individual plants spread across each site. Leaves were oven‐dried at 60°C for 72 h, and then leaves from each paired site combination were mixed together to avoid ‘home field advantage’ effects (Krna et al. 2023). We used two different‐sized mesh bags to disentangle changes in microbial decomposition from decomposition by invertebrate macrofauna. Litter bags were 10 × 10 cm and contained a total of 5 g (±0.05 g) of litter. Bags were either: (1) a ‘coarse’ nylon material mesh (4.0 mm) which enabled access by meso‐ and macrofauna that are > 1 mm in diameter on the top of the bag, with a fine nylon material mesh (1 mm) on the bottom of the bag to reduce litter loss, or (2) an entire ‘fine’ nylon mesh (1 mm) on both the top and bottom of the bag.

We randomly allocated 25 plots per site (20 plots per site for the Wellington pair) using ArcGIS. However, for ease of access, plots at SMM and Te Tapui were near existing tracks then randomly placed along each track. Six bags (three fine mesh and three coarse mesh) were placed in a closely spaced cluster on the ground at each plot (*N* = 840 bags in the study). Bags were tied together with wire to prevent bag loss, and alternated between fine and coarse mesh. Litter bags were deployed during the annual peak of invertebrate activity, from spring (October or November) 2023 until winter (late May or June) 2024, for a minimum of 186 days. Bags were left out for different durations at each paired site combination for logistical reasons, but paired sites were always collected within 6 days of each other. Collected bags were then oven dried at 60°C for 72 h and reweighed.

Three of the plots within each site had a datalogger deployed to measure temperature and relative humidity to ensure these were comparable between the pair of sites. The datalogger was shaded from direct sunlight by a piece of plastic guttering and suspended 30 cm above the forest floor. Temperature and relative humidity were recorded every 15 min. We could only use data from one logger from Waimapihi Reserve and two at Zealandia because three of the loggers malfunctioned shortly after deployment. To examine the fine‐scale differences in vegetation between sites, at each plot we recorded the percentage canopy cover (foliage above 1.3 m) using a modified method from Atkinson (1985) for each plant species in a 3 m radius around the plot point. Observors treated each canopy layer as a solid area even though there were possibly gaps between branches and estimated percentages (to the nearest 5%) of each species present, including overhanging plants in the plot area.

### 2.3 Data Analysis

We tested whether ecosanctuaries enhanced litter decomposition using a linear mixed effects model (LMM), implemented using the ‘lme4’ package (Bates et al. 2015) in R. Our response variable was the weight of decomposed litter, with site type (ecosanctuary or non‐ecosanctuary) and mesh size (fine or coarse) as fixed effects with an interaction term between them, and a random effect for plot nested within site nested within region. The nested random effect ensures that subsamples taken within each site are not treated as independent samples (Hurlbert 1984), and also accounts for the paired site design. We assessed significance by whether the 95% confidence intervals for the coefficients overlapped zero. Model‐predicted marginal means for the different fixed effect combinations were calculated using the ‘emmeans’ package. We calculated marginal and conditional *R* ^2^ values using the ‘r2_nakagawa’ function from the ‘performance’ package (Lüdecke et al. 2021). Because our sample size of sites was quite small, our approach means we can have reasonable confidence in our results in relation to our three paired sites, but caution would be needed to extend these results to other ecosanctuary sites.

We tested for fine‐scale differences in plant community composition between ecosanctuary and non‐ecosanctuary sites using permutational multivariate analysis of variance (PERMANOVA), implemented with the ‘adonis’ function from the ‘vegan’ package (Oksanen et al. 2025) in R (with 999 permutations and Bray‐Curtis distance measure). Percentage canopy scores were square‐root transformed prior to analysis, and we first checked that the assumption of multivariate homogeneity of variances was met using the ‘betadisper’ function. We also visually compared differences in composition using non‐metric multi‐dimensional scaling plots created using the ‘metaMDS’ function from the ‘vegan’ package.

All analyses were performed using R Statistical Software (v3.6.1, R Development Core Team 2019).

## 3 Results

**FIGURE 2:**
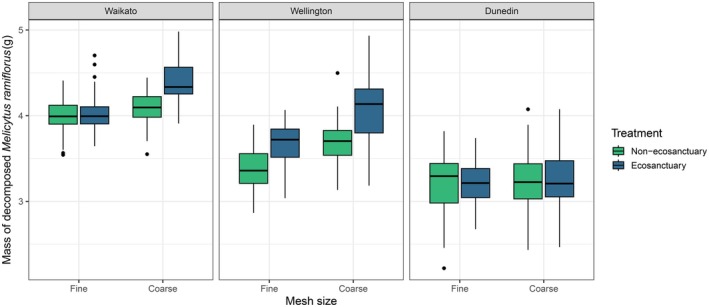
Boxplots of the mass of decomposed *Melicytus ramiflorus* litter across the three ecosanctuary (invasive mammals eradicated) and non‐ecosanctuary (minimal suppression of invasive mammals) paired sites in different regions of New Zealand.

On average, 3.9 g (SE 0.35) and 3.6 g (SE 0.24) of māhoe (from an initial 5 g) had decomposed for coarse and fine mesh treatments respectively across all three ecosanctuaries, whereas 3.6 g (SE 0.23) and 3.5 g (SE 0.24) of māhoe had decomposed on average for coarse and fine mesh treatments across all three non‐ecosanctuary sites. However, there was considerable variation in the mean mass of decomposed māhoe between the three regions (Figure 2), which was expected given their different climates and forest types (Table 1).

**FIGURE 3:**
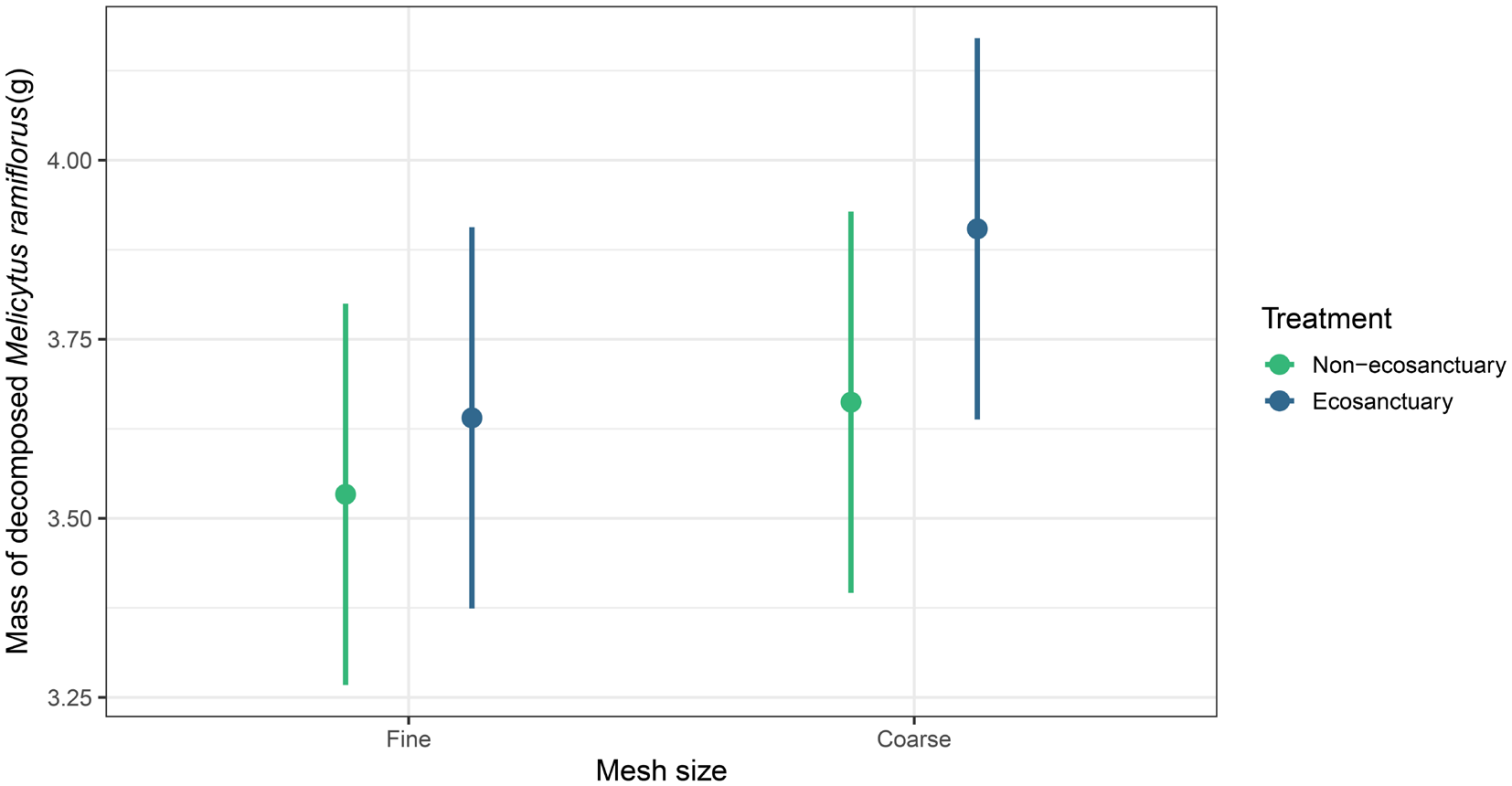
Model‐predicted marginal means (±SE) of the mass of decomposed *Melicytus ramiflorus* litter across ecosanctuary (invasive mammals eradicated) and non‐ecosanctuary (minimal suppression of invasive mammals) sites, for either fine or coarse mesh bags, from a linear mixed effects model. Litter contained in coarse mesh bags, which allowed access by meso‐ and macrofauna (> 1 mm in diameter), had faster decomposition than litter in fine mesh bags, and this effect was enhanced in ecosanctuaries compared to non‐ecosanctuary sites.

Results from our LMM demonstrated that decomposition differed between ecosanctuary and non‐ecosanctuary sites, but that this was dependent on mesh type. Coarse mesh, which permitted access by larger meso‐ and macrofauna (1–4 mm in diameter), was associated with significantly greater decomposition in ecosanctuaries than in non‐ecosanctuary sites, with the model predicting that 0.25 g more māhoe decomposed in ecosanctuaries under coarse mesh treatments. In contrast, decomposition under fine mesh did not differ significantly between site types, with the model predicting a smaller difference of 0.11 g more māhoe decomposed at ecosanctuary sites (Table 2). Figure 2 shows raw data illustrating variation in the apparent magnitude of treatment effects among the three regions, whereas Figure 3 presents the model‐predicted average treatment effect across sites, accounting for among‐site variability. Random effects explained most of the variation in the model; conditional *R* ^2^ was 0.85, while marginal *R* ^2^ was 0.06.

**TABLE 2:** Model estimates for the fixed effects (‘site type’ and ‘mesh size’) of the linear mixed effects model used to analyse the weight of decomposed *Melicytus ramiflorus* litter.

Parameters	Model estimates with 95% confidence intervals
Intercept (non‐ecosanctuary, fine mesh)	**3.53 (2.94, 4.13)**
Site type (ecosanctuary)	0.11 (−0.12, 0.34)
Mesh (coarse)	**0.13 (0.08, 0.17)**
Site type (ecosanctuary): Mesh (coarse)	**0.14 (0.08, 0.19)**

*Note:* 95% confidence intervals in parentheses. Estimates where 95% CI do not overlap zero are highlighted in bold.

**FIGURE 4:**
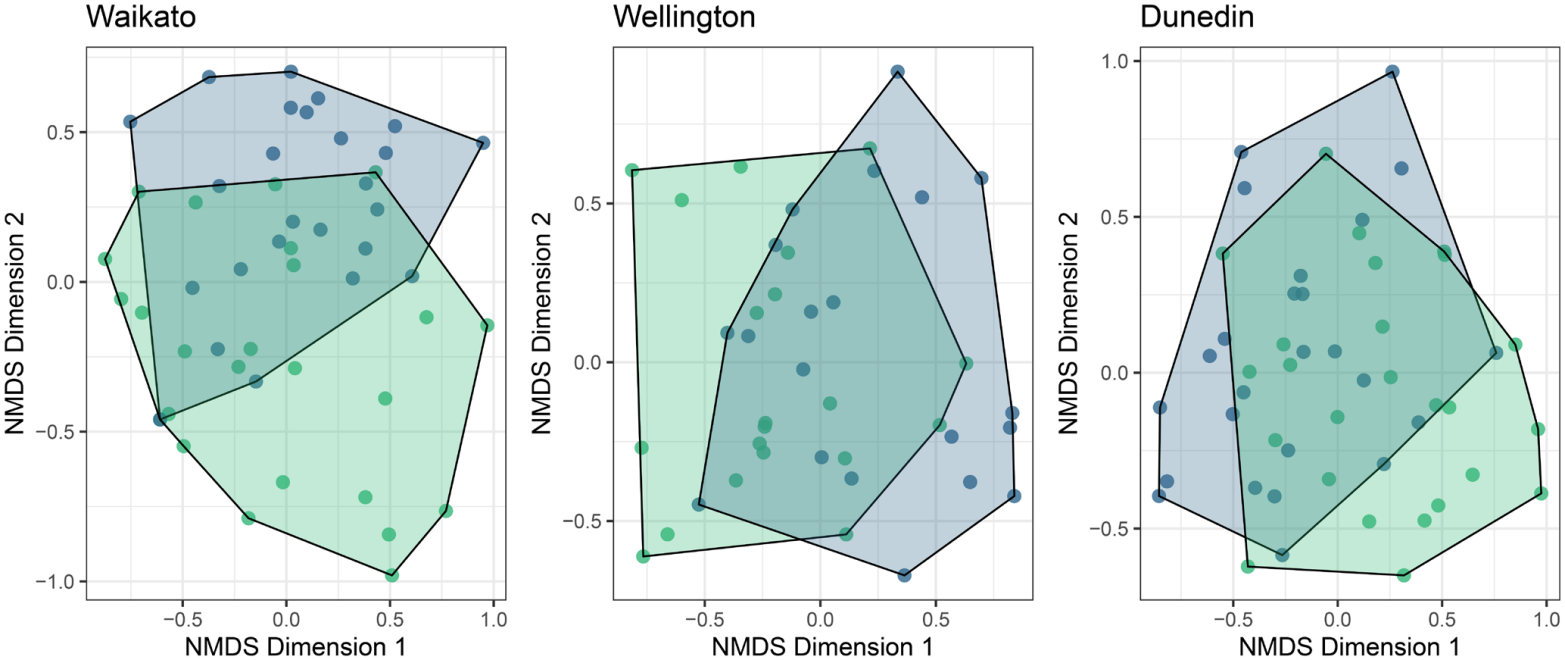
Non‐metric, multi‐dimensional scaling ordinations of plant community composition between ecosanctuaries and non‐ecosanctuaries. Blue symbols and blue shaded polygons are ecosanctuaries, and green symbols and green shaded polygons are non‐ecosanctuaries.

We found significant differences in fine‐scale plant community composition using PERMANOVA among all the ecosanctuary and non‐ecosanctuary sites (Waikato comparison: Bray‐Curtis distance measure; ADONIS: *R* ^2^ = 0.09, *p* = 0.001; Wellington comparison: Bray‐Curtis distance measure; ADONIS: *R* ^2^ = 0.08, *p* = 0.007; Dunedin comparison: Bray‐Curtis distance measure; ADONIS: *R* ^2^ = 0.08, *p* = 0.003). However, ordinations (Figure 4) created using non‐metric multi‐dimensional scaling showed large overlap in ecosanctuary or non‐ecosanctuary convex hulls for each comparison, demonstrating broad similarities.

## 4 Discussion

Restoring species and the complex interactions between them is a key aim of ecosystem restoration (Genes and Dirzo 2022). While many studies have demonstrated that eradication of invasive mammals leads to native species recovery (Binny et al. 2021; Jones et al. 2016), understanding whether this also results in the restoration of ecosystem functions has lagged. Our results demonstrate that fauna‐mediated litter decomposition was enhanced in the fenced ecosanctuaries compared to their paired non‐ecosanctuary, supporting the hypothesis that fenced ecosanctuaries, where most invasive mammals have been eradicated, restore this important ecosystem function. The results also supported our hypothesis that decomposition within fine mesh bags (which allow access by bacteria, fungi and some smaller mesofauna, but exclude larger meso‐ or macro‐invertebrates) would be similar between ecosanctuaries and non‐ecosanctuaries, but that decomposition in the coarse mesh bags (which allow access by some larger meso‐ and macro‐invertebrates) would be higher in ecosanctuaries compared to non‐ecosanctuaries.

We suggest that the enhanced litter decomposition we observed for coarse bags in ecosanctuaries was at least partly due to more abundant and diverse detritivorous invertebrates at ecosanctuaries. However, because detritivore communities were not directly sampled, this inference remains indirect. Future research integrating litterbag experiments with invertebrate community and trait analyses (e.g., functional feeding groups or body size) would strengthen mechanistic understanding. Detritivorous invertebrates shred and comminute plant litter, consume organic matter and mix soil (Mikola et al. 2002). They are a diverse group, including Isopoda, Amphipoda, Diplopoda, Collembola, Acari, Clitellata, Coleoptera and Lepidoptera. Despite the significance of detritivores to ecosystem function, they are seldom studied, and knowledge of the fauna and their interactions is poor (Swift et al. 1979; Coleman et al. 2004; Potapov 2022).

Although we did not specifically measure invertebrate diversity inside and outside the ecosanctuaries, we can draw on the results of several other studies that have. In a meta‐analysis of ecosanctuary monitoring data, Binny et al. (2021) found that certain invertebrate orders benefited from mammal removal, including important detritovore groups such as Isopoda. Watts et al. (2014) found that there were ‘winners’ and ‘losers’ in the beetle community at Zealandia 7 years after mammal eradication (excluding mice), with larger taxa benefiting from mammal eradication. Other New Zealand studies comparing ground‐dwelling invertebrate communities inside and outside fenced areas have detected some significant differences in invertebrate community composition, but trophic groups showed varied responses to mammal eradication. For example, at SMM, analysis of the beetle community from a functional trait perspective showed that large, predatory and native beetles differed the most between inside and outside the fenced southern exclosure with no mammals over some years between 2004 and 2013 (Watts et al. 2020). It was also apparent that one species (*Ctenognathus adamsi*) of predatory Carabidae was 20 times more abundant inside Zealandia than outside the reserve (Vergara et al. 2021). In contrast, Chen et al. (2022) found no evidence to suggest that beetles belonging to various trophic groups varied inside and outside the fence at Orokonui. However, further analysis showed that trophic composition inside and outside the fence was dependent on the vegetation composition of the habitat, with more fungivores/decomposers being observed inside the fence for grassy habitats (Chen et al. 2022). Fewer studies have been conducted outside of New Zealand. However, using novel long‐term exclusion experiments in Australia, Gibb et al. (2021) found that mammals had direct negative effects on scorpions and spiders, and that spider community composition changed following mammal exclusion.

Invertebrate community responses in ecosanctuaries have been idiosyncratic and could be limited by increased predation pressure from recovering bird populations and the presence of mice. Although mice are known to persist in several ecosanctuaries (including two of our three study ecosanctuaries) and exert strong negative effects on ground‐dwelling invertebrates, records of mouse presence or activity were not included in our analysis as we did not have sufficient sites to model another covariate. We acknowledge this limitation, as variation in mouse abundance among sites may have influenced decomposition and potentially confounded treatment effects attributed to ecosanctuary status. For example, in a recent study of the impacts of mice alone on biodiversity at SMM, Watts et al. (2022) found strong evidence that mice reduced the abundance of ground‐dwelling invertebrates (especially caterpillars, spiders, wētā and beetles), and reduced the mean body size of some taxa. In addition, earthworm abundance, biomass and species richness increased with a decreasing mouse population in one study block. Therefore, an important next step would be to experimentally test the effects of mouse exclusion on decomposer communities and decomposition rates.

More generally, most research aimed at understanding invertebrate communities inside and outside New Zealand ecosanctuaries has focused on the response of beetles. We recommend that future research sample other important detritivore groups, such as Isopoda, Amphipoda, Diplopoda, Collembola and Clitellata, in tandem with litterbags. We would expect larger detritivorous taxa, such as earthworms, to respond the strongest to mammal removal within ecosanctuaries. Invertebrate samples could be analysed in relation to specific traits relating to detritivorous invertebrates (e.g., feeding mode [including shredders] and size). Alternatively, DNA metabarcoding provides an opportunity to measure the DNA of litter organisms feeding on litter (Lopes et al. 2021) in bags to investigate what the main decomposers are and whether the composition of these communities differs between ecosanctuaries and non‐ecosanctuaries.

The removal of browsers (especially ungulates such as deer and goats, but also rabbits) from the ecosanctuaries is also likely to have resulted in complex changes to above‐ and below‐ground processes that could have underpinned our results. Excluding browsing mammals enhances the density and diversity of understorey vegetation and promotes the regeneration of palatable plant species with high litter quality, thereby altering the quantity and quality of litterfall (Wardle, Barker, Yeates, et al. 2001; Wardle et al. 2002). Although we attempted to sample the same dominant vegetation type between the ecosanctuary and non‐ecosanctuary sites, our vegetation analyses demonstrate that there were fine‐scale differences between the paired sites, potentially because of the removal of browsers. Similarly, Blick et al. (2008) found in the decade following mammal exclusion at Zealandia that complex successional changes in the vegetation had occurred, including seedling abundance increasing and plant species composition changing markedly through time. Tanentzap and Lloyd (2017) also observed a higher abundance of mammal‐sensitive plant species following mammal eradication at Orokonui. While we did not measure litter composition and quality, it seems likely this would be enhanced at ecosanctuaries and may have altered the below‐ground processes.

Invasive browsing mammals have consistent impacts on vegetation properties, but their indirect effects on the decomposer communities may be more idiosyncratic (Bardgett and Wardle 2010). In particular, microbes and microfauna respond inconsistently to browsing mammal exclusion, whereas larger meso‐ and macro‐fauna often respond positively (Wardle, Barker, Yeates, et al. 2001), potentially because these latter groups are more affected by physical disturbance such as trampling. Our results—that decomposition by smaller‐bodied decomposers was unaffected by ecosanctuary status, whereas decomposition by larger meso‐fauna was improved—support Wardle, Barker, Yeates, et al.'s (2001) findings.

Although our research demonstrated that ecosanctuaries had greater fauna‐mediated decomposition compared to non‐ecosanctuaries, there was some variability in this response between the site pairs. While we addressed issues associated with low replication as robustly as possible, limited site numbers inevitably constrain the strength and generality of the conclusions that can be drawn from this study. The two North Island ecosanctuaries showed strong effects of treatment, whereas the Dunedin paired sites did not seem to differ in decomposition rates for either fine or coarse mesh. There could be several reasons for this. The duration since ecosanctuary establishment may have an effect, as Orokonui has been an ecosanctuary for the shortest amount of time (since 2007). However, this is only 1 year shorter than SMM so is unlikely to be a significant cause. The differences observed between the North Island and South Island paired sites could be caused by an interaction between temperature and mammal removal. It is possible that in warmer temperatures invertebrate fauna and the processes they mediate can recover faster because it is, generally, a more productive system. Related to this idea, there could be a lower biomass of decomposer communities or litter‐dwelling invertebrates in cold South Island ecosystems compared to warmer North Island forests. For example, ground‐dwelling invertebrate biomass has been shown to decline with decreasing temperature across elevational gradients in New Zealand (Schlesselmann et al. 2023; Carpenter et al. 2022).

Conservation practitioners wanting to enhance decomposition processes further could consider ‘rewilding’ decomposer communities, defined here as ‘the intentional restoration of ecological processes through the reintroduction or recovery of missing biota and their interactions’ (Perino et al. 2019). In this context, rewilding could be implemented by transplanting litter and soil from more species‐rich, old‐growth forests to re‐establish diverse decomposer assemblages and functional processes. This practice was recently tested in Australia, where transplantations from species‐rich remnant sites to isolated revegetated farmland sites led to faster decomposition rates at restoration sites due to a greater abundance of invertebrate detritivores (Contos et al. 2024). However, there are risks associated with litter transplants that need to be considered before undertaking rewilding, such as the spread of invasive species or pathogens. We also recommend leaving woody detritus (e.g., fallen trees) in situ to support diverse decomposer communities (Ulyshen 2016).

New Zealand's fauna evolved in the absence of mammals (with the exception of three bat species), leading invertebrates to take on ecological roles typically occupied by mammals elsewhere (e.g., Duthie et al. 2006), making them crucial for many terrestrial ecosystem processes. However, it can be difficult to identify the pre‐mammal baselines that restoration efforts might aim to replicate. Watts et al. (2019) examined the attributes of the beetle community at SMM and compared them with the fossil beetle assemblage from forested North Island sites prior to the introduction of mammals and found no difference between the trophic composition and dispersal ability of taxa within the beetle communities sampled, although several large flightless ground‐dwelling weevil species (e.g., *Tymbopiptus valeas* and *Anagotus* sp. 1) had gone extinct. Further research into prehistoric baselines of invertebrate communities would be invaluable for providing benchmarks for assessing restoration outcomes. Finally, when considering pre‐human baselines, it is important to recognise that New Zealand's ecosystems were not only free of most mammals but also contained more diverse and abundant bird communities, including keystone herbivores and insectivores, such as moa and kiwi (Holdaway and Worthy 1997; Wood 2023). These species likely had major impacts on litter dynamics and invertebrate communities. Hence, restoration baselines should account both for the loss of native avian ecosystem engineers and the introduction of new mammalian ones. We also note the relevance of the keystone species framework (Payton et al. 2002) to our research, as it contextualises invasive mammal impacts and the cascading recovery of ecosystem processes following their removal.

Recent studies have shown that fenced ecosanctuaries are restoring avian mutualisms such as seed dispersal and pollination (e.g., Bombaci et al. 2021; Iles and Kelly 2014; Van Etten et al. 2018), and our findings suggest that recovery of litter decomposition is also occurring. To strengthen inference in future work, we recommend (1) conducting studies across a larger number of ecosanctuary and non‐ecosanctuary sites to improve replication and capture broader environmental variability, and (2) implementing Before–After–Control–Impact (BACI) study designs wherever new ecosanctuaries are planned, enabling stronger causal attribution of observed changes in ecosystem function to mammal eradication. The obvious next step is to assess whether some of the other essential ecosystem functions carried out by invertebrates, such as pollination, are also being restored. Encouragingly, Liang et al. (2022) demonstrated that suppression of invasive rats, mice, ants and wasps increased pollinator visitation rates in Hawaiʻi, with greater effects predicted for full predator eradication. Further analysis of other ecosystem functions, across both invasive mammal suppression and eradication regimes, will fill critical gaps in our understanding of how conservation interventions lead to ecosystem recovery.

## Author Contributions

**Corinne Watts:** conceptualization (equal), data curation (lead), funding acquisition (lead), investigation (equal), methodology (equal), project administration (lead), writing – original draft (equal). **Christopher K. Woolley:** data curation (equal), investigation (equal), methodology (equal), writing – review and editing (equal). **James Arbuckle:** data curation (equal), investigation (equal), methodology (equal), writing – review and editing (equal). **Scott Bartlam:** data curation (equal), investigation (equal), methodology (equal), writing – review and editing (equal). **Joanna K. Carpenter:** conceptualization (equal), formal analysis (lead), investigation (equal), methodology (equal), writing – original draft (equal).

## Funding

This research was funded by the Strategic Science Investment Fund for Crown Research Institutes from the Ministry of Business, Innovation and Employment's Science and Innovation group.

## Conflicts of Interest

The authors declare no conflicts of interest.

## Data Availability

Data used in this study are available from the Manaaki Whenua—Landcare Research DataStore (https://doi.org/10.7931/sgnf‐np74).
